# Development of a Biosensor for Environmental Monitoring Based on Microalgae Immobilized in Silica Hydrogels

**DOI:** 10.3390/s121216879

**Published:** 2012-12-06

**Authors:** Yannis Ferro, Mercedes Perullini, Matias Jobbagy, Sara A. Bilmes, Claude Durrieu

**Affiliations:** 1 Université de Lyon, ENTPE, CNRS, UMR5023 LEHNA, Rue Maurice Audin, 69518 Vaulx-en-Velin, France; E-Mail: claude.durrieu@entpe.fr; 2 INQUIMAE-DQIAQF, Universidad de Buenos Aires, Facultad de Ciencias Exactas y Naturales, Ciudad Universitaria Pab. II, C1428EHA Buenos Aires, Argentina; E-Mails: jobbag@qi.fcen.uba.ar (M.J.); sarabil@qi.fcen.uba.ar (S.A.B.)

**Keywords:** biosensor, algae, chlorophyll fluorescence, DCMU, atrazine, sol-gel, herbicides

## Abstract

A new biosensor was designed for the assessment of aquatic environment quality. Three microalgae were used as toxicity bioindicators: *Chlorella vulgaris*, *Pseudokirchneriella subcapitata* and *Chlamydomonas reinhardtii.* These microalgae were immobilized in alginate and silica hydrogels in a two step procedure. After studying the growth rate of entrapped cells, chlorophyll fluorescence was measured after exposure to (3-(3,4-dichlorophenyl)-1,1-dimethylurea) (DCMU) and various concentrations of the common herbicide atrazine. Microalgae are very sensitive to herbicides and detection of fluorescence enhancement with very good efficiency was realized. The best detection limit was 0.1 μM, obtained with the strain *C. reinhardtii* after 40 minutes of exposure.

## 1. Introduction

Deterioration of the aquatic environment by pollutants is an important problem. Among all of them, heavy metals and herbicides constitute a priority preoccupation: they are frequently found in surface and ground waters and are harmful to aquatic organisms [1–5]. The need for convenient, quick and reliable methods to assess pollutant toxicity is more essential than ever. Environmental monitoring of pollutants with automatic systems applied online and allowing rapid response is one of the best ways to control the quality of the environment. Real time analysis offers the advantage of detecting rapidly the presence of pollutants before they cause any damage. Such a strategy is only possible through biosensors [6].

Photosystem II (PSII)-based biosensors are reported to be able to detect herbicides in the environment [7]. Various herbicides, including the urea, triazine, and phenolic based herbicides, target the vegetal PSII. These substances inhibit photosynthetic electron flow by blocking the PSII quinone binding site and thus modify chlorophyll fluorescence. Many biosensors based on algal chlorophyll fluorescence measurement were developed and have demonstrated the suitability of this technique for herbicide detection [8–11].

An important point in the design of algal biosensors is the question of cell-binding. One of the most commonly employed procedures, based on the immobilization of microalgae within calcium crosslinked alginate beads is intrinsically biocompatible [12–16], but such matrices are disrupted in water as well as by alginate biodegradation. In the last decade, there have been huge improvements in the development of biocompatible synthetic routes to entrap biological entities within pure inorganic matrices [17–23]. Among them, routes based on sol–gel chemistry [24] are unique in offering the necessary mild conditions for building composite materials based on robust silica hydrogels and biological entities where the bio-activity gives rise to a wide range of sensing possibilities [25–27].

The design of biosensors based on direct silica encapsulation is limited by the restricted cell viability, but recent work has demonstrated the possibility of cell division inside inorganic matrices by means of a two-step encapsulation procedure based on sol–gel chemistry [28–30]. This strategy expands the range of possible applications as cells can not only be entrapped within silica hydrogels, but are also able to grow inside, even for periods of months [31]. In addition, the inherent homogeneous and mesoporous texture of the silica hydrogel provides a shield preventing the release of entrapped cells, as well as the contamination of the inner culture by exogenous strains. Moreover, in previous works we have demonstrated the long-term viability of algal cells encapsulated in an alginate-silica hybrid matrix [32].

In this paper, translucent alginate-silica matrices obtained by a sol-gel process have been investigated for entrapment of three algal strains in order to improve biosensor fabrication. After a biocompatibility assay of the inorganic host material, the effects of algal immobilization were tested on their ability to determine herbicides.

## 2. Experimental Section

### 2.1. Cell Cultures

*Chlorella vulgaris*, *Pseudokirchneriella subcapitata* and *Chlamydomonas reinhardtii* were used. Algal strains were purchased from The Culture Collection of Algae and Protozoa (Cumbria, United Kingdom). *C. vulgaris* and *P. subcapitata* were grown in Lefebvre-Czarda medium [33] whereas *C. reinhardtii* was grown in trisacetate phosphate (TAP) medium [34] and were transplanted weekly under sterile conditions (autoclaving 20 minutes, 130 °C, 1.3 bars). Algae were maintained in a nycthemeral cycle of 16 hours of illumination at 5,000 lux and eight hours of darkness.

### 2.2. Immobilization Method

**Figure 1.**
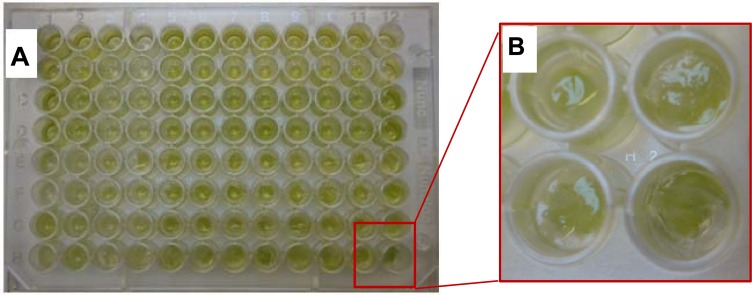
Photograph of the biosensor with *C. reinhardti* (**A**); Zoom on wells with two step-immobilized algae (**B**).

In a two-step procedure, cells were previously immobilized in Ca(II)-alginate beads which was subsequently trapped in the inorganic matrix, avoiding any harmful contact of cells with any silica precursors (Figure 1). This procedure based on inexpensive silica precursors has already been employed for yeast (*Saccharomyces cerevisiae*), bacteria (*Escherichia coli* and *Bacillus subtilis*), algae (*Chlorella vulgaris*), fungi (*Stereum hirsutum*) and plant cells (BY2 tobacco and carrot phloematic tissue) [31].

#### 2.2.1. Alginate Beads and Gels

Formation of alginate beads was performed by dropwise addition of a 2% (w/w) Na(I)-alginate cells suspension in a 0.1 M CaCl_2_ solution. After 10 min stirring, beads of about 3 mm diameter were easily collected by filtration. The Ca(II)-alginate polymer prevents cell contact with synthesis precursors.

Formation of alginate gel beads in 96 multiwells plate was performed by stirring a 50 μL aliquot of cells suspended with 50 μL of Tris-HCl buffer (10 mM, pH = 7.5) and 100 μL 2% Na(I)-alginate (resp. 100, 100 and 200 μL for 48 multiwell plates). This mixture was introduced in the cavities of the sample holder. Alginate cross-linking was built by immersing the sample holder into a 0.1 M CaCl_2_ solution for 10 minutes.

#### 2.2.2. Silica Hydrogels Synthesis

The second step of the immobilization procedure consists of a silicate (sodium silicate, Riedel-de Haën; NaOH 10%, SiO_2_ 27%) sol-gel process in the presence of commercial silica nanoparticles (LUDOX HS-40, 40% in water, obtained from Aldrich), leading to a nanoporous monolithic structure. Monoliths were prepared at room temperature by mixing volumes of the different precursor solutions to obtain a SiO_2_ water molar relation of 3.8% with a fixed proportion of polymeric to particulate silica precursors (1:3) at constant pH = 6.5, adjusted with HCl.

### 2.3. Growth Rate of Algae Entrapped within Silicate-Ludox-Based Hydrogels

To evaluate the degree in which the development of algae is affected by the encapsulation in alginate and silica, the algal growth rate as a function of entrapment method was measured. The algal growth inside the voids was studied for individual cavities after 3 days of culture in LC liquid medium. At the initial time, calcium alginate beads with a content of 10^−4^ cells/mL were dispensed into 1.5 mL tubes (Eppendorf), and the silica encapsulation procedure was performed. After 3 days, the silica hydrogel was removed and samples were exposed to 0.05% potassium citrate to solubilize the Ca(II)-alginate beads. The total number of cells inside individual cavities was determined by counting cells in a Mallassez counting chamber. To analyze cellular growth, the percentage of inhibition (I) is calculated using the equation:

$$I=\frac{\mu_{c}-\mu_{i}}{\mu_{c}}100$$

where μ_i_ is the growth rate for test batch i (entrapped cells) and μ_c_ is the mean growth rate for the control batch (free cells). Test and control batches were run in triplicate.

### 2.4. Biosensor Performance

The herbicides (3-(3,4-dichlorophenyl)-1,1-dimethylurea) (DCMU, 4 mg/L) and atrazine (10 μM to 0.001 μM) were used as examples of anti-PSII herbicides to evaluate the performance of the fluorescence biosensor (Table 1).

Response of the algal biosensor was obtained from the chlorophyll fluorescence emission at 682 nm under a 469 nm excitation light with a spectrofluorimeter (Fluostar, BMG). The biosensor response to herbicide was determined by the fluorescence enhancement (E) as soon as a defined amount of herbicide is added to the sample (the herbicide is dissolved in distilled water and added to free and entrapped algae).

For herbicides which inhibit PSII, the enhancement can be determined after 40 minutes using the flowing equation:

$$E=\frac{F_{a}-F_{b}}{F_{b}}100$$

where F_b_ is the fluorescence for a test batch before contact with the herbicide and F_a_ is the fluorescence for the same test batch after the herbicide exposure.

All assays were undertaken with a thickness of silica layer around 1.9 mm and with the following cellular concentrations: [CV] ≈ 3.3 10^6^ cells/mL; [PS] ≈ 1.4 10^6^ cells/mL; [CR] ≈ 1.4 10^6^ cells/mL.

## 3. Results and Discussion

### 3.1. Growth Rate of Entrapped Algae

In the design of biosensors with encapsulated photosynthetic cells, as well as for the design of devices based on optical detection systems it is mandatory to minimize the attenuance of the host matrix in the visible region [35]. The hydrogel properties depend on its microstructure, which can be controlled by sol–gel synthesis variables. In particular, optical properties of silica hosts obtained *via* a mixed aqueous route based on sodium silicate–silica nanoparticle mixtures can be improved by increasing the relative content of molecular precursors over colloidal silica [36]. By means of the two-step procedure and due to the protection provided by the Ca(II)-alginate pre-encapsulation, silica synthesis can be attempted under more cytotoxic conditions, allowing the use of a higher ionic strength than possible in one-pot encapsulation procedures. In a previous work, we investigated the degree to which the scattering of visible light affects the encapsulated algae growth rate, analyzing the number of *Chlorella vulgaris* cells developed in voids created inside silicate-Ludox hydrogels as a function of gel thickness [32]. Following the same encapsulation procedure as described in the previous section, except for the proportion of polymeric to particulate silica precursors, which was varied in the range 1:3 to 1:5, it was found that the growth of *C. vulgaris* was affected by both the attenuance of the matrix and the host thickness. However, for the matrix with higher polymeric precursor content, the growth rate was unaffected, even for gel thicknesses up to 4.0 mm.

**Figure 2.**
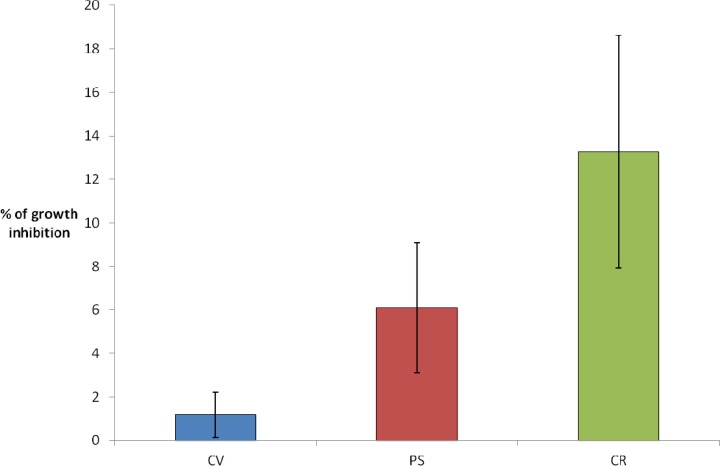
% of growth inhibition after the two steps immobilized algae (silica layer = 1.9 mm) in comparison with free algae (none immobilized). Three algal strains are compared: *C. vulgaris* (CV, in blue), *P. subcapitata* (PS, in red) and *C. reinhardtii* (CR, in green).

In the present study, the optimized ratio of polymeric to particulate silica precursors (*i.e.*, 1:3) was investigated for the two-step encapsulation of *C. reinhardtii* (CR), *P. subcapitata* (PS) and *C. vulgaris* (CV). Silica hydrogel thickness was fixed at 1.9 mm, since in the previous study, regardless of the optical quality of the silica matrix, no detriment in CV growth rate was found for this silica thickness. At the initial time, calcium alginate beads with a content of 10^4^ cells/mL were dispensed into acrylic molds, and the silica encapsulation was performed. The algal growth inside the voids was studied for individual cavities after 3 days of culture in AFNOR liquid medium. As shown in Figure 2 and in agreement with previous results, [32] CV growth rate was almost unaffected (growth inhibition ≈ 2%). For the other algal species, we could see a slight difference of sensibility, although in the three cases these results confirm the biocompatibility of the two-step procedure immobilization with a relatively low growth inhibition (6% and 13% for PS and CR, respectively).

### 3.2. Algal Fluorescence Measurements

#### 3.2.1. Immobilization Effect

Since algal cells are entrapped in an insoluble support, they are no longer in their natural environment. It is also necessary to assess their activity in the presence of various compounds added during the immobilization process.

**Figure 3.**
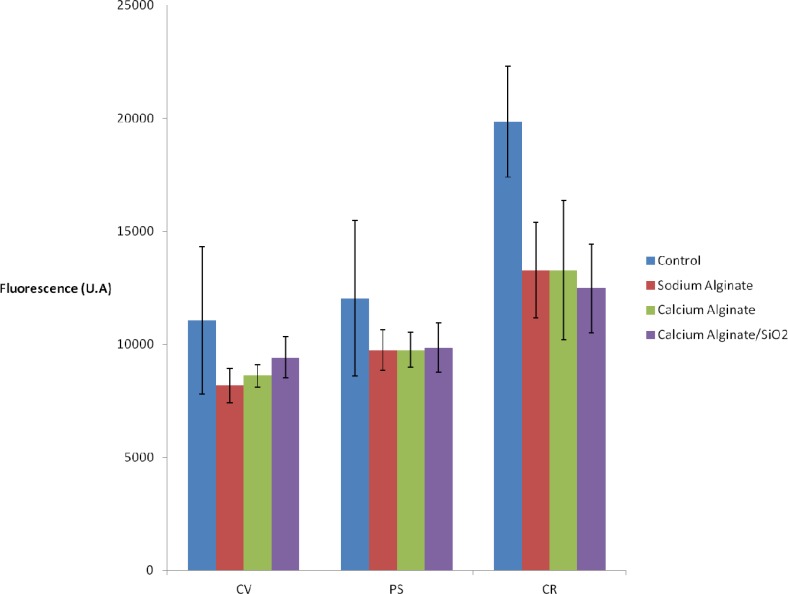
Algal chlorophyll fluorescence 1 h after the deposition in microplate: in free culture medium (control, in blue), in culture medium added with 2% Na(I)-alginate (sodium alginate, in red), encapsulated in Ca(II)-alginate (calcium alginate, in green), and after the two steps immobilization procedure (calcium alginate/SiO_2_, in purple). Three algal strains were used: *C. vulgaris* (CV), *P. subcapitata* (PS) and *C. reinhardtii* (CR).

Differences observed according to the immobilization method and the studied strains are slight (Figure 3). A small decrease of the fluorescence is observed when 2% Na(I)-alginate is added to the culture medium. The fact of immobilizing algae (in calcium alginate or in calcium alginate inside the silica matrix) does not lead to further loss of signal. Thus, the activity of algal cells entrapped within Ca(II)-alginate matrices—a standard procedure for biosensor applications—is not affected by our encapsulation procedure. On the other hand, this provides new evidence that light scattering by the silica matrix is negligible.

#### 3.2.2. Effect of DCMU on Chlorophyll Fluorescence

**Figure 4.**
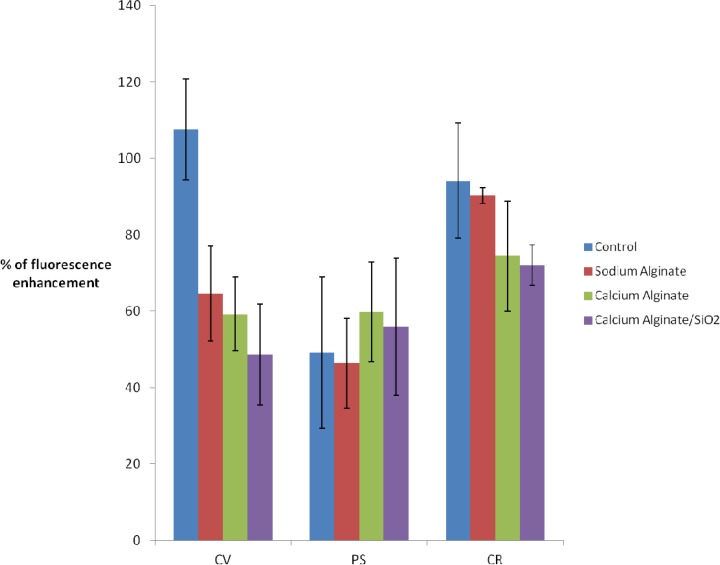
% of chlorophyll fluorescence enhancement after 40 min DCMU (4 mg/L) exposure according to entrapment method and algal strain: in free culture medium (control, in blue), in culture medium added with 2% Na(I)-alginate (sodium alginate, in red), encapsulated in Ca(II)-alginate (calcium alginate, in green), and after the two steps immobilization procedure (calcium alginate/SiO_2_, in purple). Three algal strains were used: *C. vulgaris* (CV), *P. subcapitata* (PS) and *C. reinhardtii* (CR).

According to Figure 4, there is a 40% decrease in fluorescence enhancement when *C. vulgaris* is cultured with the addition of 2% Na(I)-alginate. This could be partially attributed to the loss of chlorophyll fluorescence observed in the absence of DCMU (see Figure 3). However, the fact of immobilizing algae [in Ca(II)-alginate or in Ca(II) alginate/silica matrix] does not lead to further enhancement losses.

Analyzing the *P.subcapitata* response to DCMU exposure, the fluorescence enhancement for free PS and PS suspended in alginate, Ca(II)-alginate and hybrid Ca(II)-alginate and silica, does not show significant differences (in all cases enhancement is around 50% ± 20%).

Finally, for the *C. reinhardtii* strain, after sol-gel entrapment fluorescence enhancement is strong, with a small variability (75% ±5) and close to control (free algae: 90% ±10).

These results show the ability of the entrapped microalgae biosensor to detect DCMU with accuracy comparable to that obtained with free algae. The next step is the determination of the detection limits (time and concentration) and the possibility of sensing another herbicide such as the atrazine.

#### 3.2.3. Effect of Atrazine on Chlorophyll Fluorescence

**Figure 5.**
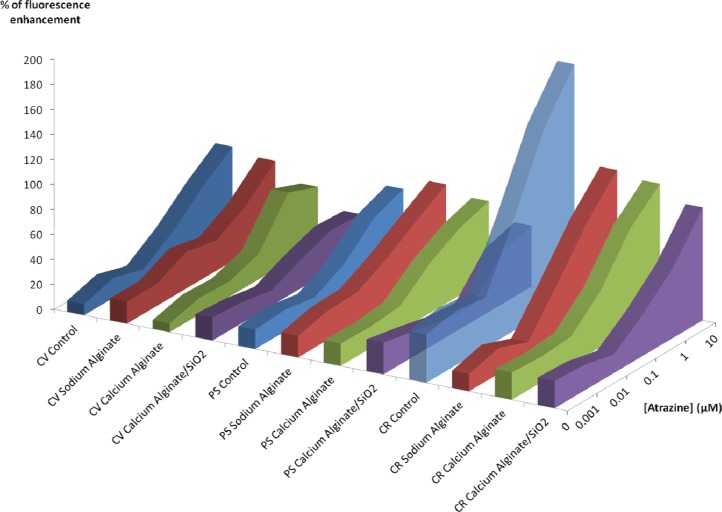
% of chlorophyll fluorescence enhancement after 40 min atrazine exposure according to entrapment method and algal strain. In free culture medium (control, in blue), in culture medium added with 2% Na(I)-alginate (sodium alginate, in red), encapsulated in Ca(II)-alginate (calcium alginate, in green), and with the two steps immobilization procedure (calcium alginate/SiO_2_, in purple). Three algal strains were used: *C. vulgaris* (CV), *P. subcapitata* (PS) and *C. reinhardtii* (CR).

Figure 5 summarizes the results obtained with the three algal strains after atrazine exposure. After an exposure of 40 minutes on *C. vulgaris*, we obtained a detection limit of 0.1 μM for all the immobilization methods, indicating that at this concentration, there is no difference between different encapsulation methods. Upon increasing the atrazine concentration, the chlorophyll fluorescence enhancement of silica entrapped algae does not increase, contrary to what is observed for the free algae and for the other encapsulation methods, which effectively show a dose response.

With *P. subcapitata,* the detection limit after 40 minutes is still 0.1 μM for free PS, PS suspended in alginate, Ca(II)-alginate, but it is found to be higher for PS encapsulated in Ca(II)-alginate-silica (1 μM).

In the case of *C. reinhardtii,* the loss of sensibility between free algae and encapsulated ones is very strong, regardless of the entrapment method, but compared to the other strains the CR are the most sensitive. For example, on algae entrapped within silica hydrogels after a 40 minutes exposure to 1 μM, the fluorescence enhancement is 57% for CR, 42% for PS and 32% for CV. Furthermore, the limit of detection after 40 mn is the lowest with *C. reinhardtii*: 0.1 μM for all tests batches, even those with Ca(II)-alginate-silica.

Table 2 summarizes the results obtained with the two steps encapsulation procedure after atrazine exposure to different contact times. When there is no pollutant (0 μM, 1st column), there is a fluorescence enhancement after 40 minutes. This could be explained by the growth of the culture. The time of exposure is important and the longer it is, the more is the fluorescence, but between 5 hours and 24 hours, with 10 μM pollutant exposure, we observe a decrease of the fluorescence enhancement for *C. reinhardtii.* This is attributed to an important toxicity of atrazine and the subsequent cellular mortality. Finally, the choice of the strain is an important factor for the sensitivity of the biosensor. For example after 5 h with 1 μM pollutant exposure, the fluorescence enhancement is around 71% with CV, 49% with PS and 92% with CR. This is a general result and our results allow us to sort algal strain according to their herbicide sensitivity in the following order: CR > CV > PS.

## 4. Conclusions

In this study, we demonstrate the feasibility of using an inorganic translucent hydrogel to construct an optical biosensor with immobilized algal cells. According to our results and in agreement with former studies [27], the sol-gel immobilization in two steps shows a high biocompatibility with green algae; this encapsulation method is easy to perform and allows one to obtain a matrix with good optical and mechanical performance. Furthermore, the polymeric and particulate silica precursors used in this synthesis are low cost reagents, which is stimulating from the point of view of the scaling up in future applications. The activity of algae whole cells immobilized within the silica hydrogel can be assessed from chlorophyll fluorescence measurements. The translucence of the structure allowed the algal layer to be placed directly in contact with the optical fibers to produce a fluorescence emission detectable by a fluorometer. In addition, this whole-cell reagentless biosensor is economically very interesting because it requires neither substrate nor fluorescent indicator to monitor herbicide levels in the environment.

The use of this entrapment technique is facilitated by the optical transparency, mechanical stability and chemical inertness of the silica host. Optical properties are of crucial importance for both the growing of photosynthetic algal cells inside the biosensing devices and for the detection method based on the determination of the chlorophyll fluorescence enhancement. Since the porosity of the silica matrix (in the range of mesopores) can be tuned by the sol-gel synthesis method used, the transport properties of the host material can be regulated in order to allow free diffusion of aqueous pollutants to make them accessible to algal cells. This technique has been applied to the determination of DCMU and atrazine, as examples of herbicides that are known to inhibit the algal PSII. Among the algal strains tested, *Chlamydomonas reinhardtii* is the most sensitive specie for atrazine determination, with a limit of detection of 0.1 μM after 40 minutes of exposure.

This optical algal biosensor could be used as an early-warning device to detect those pollutants in effluents. Performance and optimization studies with other herbicides are under way and the results will be compared to other analytical techniques. Before an *in situ* implementation the limits of detection have to be improved. Concentrations of DCMU and atrazine in controlled effluents are around 0.1 and 10 μg/L and European Environmental Quality Standards (EEQS) are 0.2 μg/L for DCMU and 0.6 μg/L for atrazine [3,37,38].

On previous papers, we have shown that the use of *C. vulgaris* cells as a bioreceptor allowed the determination of the antiphotosystem II (PSII) herbicides group which target the algal PSII fluorescence [10]. Under the same conditions ([*C. vulgaris*] = 1.5 × 10^6^ cells/mL and phosphate buffer pH 7.0) the limit of detection of atrazine was 1 μg/L (5 × 10^−3^ μM) with 1% fluorescence increase. These results were obtained with a high sensitivity fluorometer equipped with single photon counting capabilities (the Spex Fluorolog 2). We believe that using a more accurate fluorometer and by improving the immobilization method to decrease the variability, we could further reduce these detection limits.

## Figures and Tables

**Table 1.** Experimental design on the microplate Co: control (no herbicide), C1 = 0.001 μM, C2= 0.01 μM, C3=0.1 μM, C4=1 μM and C5 = 10 μM. in free culture medium (in blue), in culture medium added with 2% Na(I)-alginate (sodium alginate, in red), encapsulated in Ca(II)-alginate (calcium alginate, in green), and after the two steps immobilization procedure (calcium alginate/SiO_2_, in purple).

	1	2	3	4	5	6	7	8	9	10	11	12
A	Co	Co	C1	C1	C2	C2	C3	C3	C4	C4	C5	C5
B	Co	Co	C1	C1	C2	C2	C3	C3	C4	C4	C5	C5
C	Co	Co	C1	C1	C2	C2	C3	C3	C4	C4	C5	C5
D	Co	Co	C1	C1	C2	C2	C3	C3	C4	C4	C5	C5
E	Co	Co	C1	C1	C2	C2	C3	C3	C4	C4	C5	C5
F	Co	Co	C1	C1	C2	C2	C3	C3	C4	C4	C5	C5
G	Co	Co	C1	C1	C2	C2	C3	C3	C4	C4	C5	C5
H	Co	Co	C1	C1	C2	C2	C3	C3	C4	C4	C5	C5

**Table 2.** % of chlorophyll fluorescence enhancement after contact with atrazine (0 to 10 μM) on algae entrapped within silica hydrogels according to different contact times (40 minutes, 5 hours and 24 hours). Three algal strains were used: *C. vulgaris* (CV), *P. subcapitata* (PS) and *C. reinhardtii* (CR).

**% of Fluorescence Enhancement**		**[Atrazine] (μM)**					

**Algal Strain**	**Contact Time**	**0**	**0.001**	**0.01**	**0.1**	**1**	**10**

**CV**	**40 min**	18.5 ± 2.73	16.65 ± 11.95	12.29 ± 2.93	23.44 ± 8.9	32.17 ± 7.19	30.95 ± 1.42
	**5 h**	24.83 ± 4.39	23.24 ± 4.03	25.92 ± 3.94	46.89 ± 8.7	71.49 ± 11.22	74.11 ± 4.69
	**24 h**	76.72 ± 1.82	30.93 ± 14.17	31.22 ± 3.78	60.55 ± 2.23	91.78 ± 13.62	77.86 ± 3.68

**PS**	**40 min**	25.37 ± 5.09	20.44 ± 8.69	14.85 ± 10.01	12.39 ± 8.60	41.71 ± 6.40	48.77 ± 11.38
	**5 h**	19.02 ± 2.69	21.36 ± 8.60	17.37 ± 9.93	22.11 ± 10.63	48.97 ± 1.54	62.27 ± 4.3
	**24 h**	20.42 ± 1.02	25.47 ± 8.17	23.04 ± 10.42	29.57 ± 11.40	68.13 ± 2.46	48.68 ± 2.01

**CR**	**40 min**	21.91 ± 5.78	20.71 ± 11.15	15.02 ± 3.28	33.64 ± 20.89	56.52 ± 23.77	90.49 ± 7.1
	**5 h**	28.91 ± 3.35	32.07 ± 12.68	26.41 ± 2.13	58.75 ± 26.79	91.68 ± 25.08	136.97 ± 9.37
	**24 h**	33.44 ± 5.65	37.49 ± 13.61	28.17 ± 1.67	62.7 ± 23.82	71.53 ± 25.95	85.12 ± 15.2

## References

[1] Hanke I., Wittmer I., Bischofberger S., Stamm C., Singer H. (2010). Relevance of urban glyphosate use for surface water quality. Chemosphere.

[2] Reilly T.J., Smalling K.L., Orlando J.L., Kuivila K.M. (2012). Occurrence of boscalid and other selected fungicides in surface water and groundwater in three targeted use areas in the United States. Chemosphere.

[3] Guzzella L., Pozzoni F., Giuliano G. (2006). Herbicide contamination of surficial groundwater in Northern Italy. Environ. Pollut.

[4] Davis A.P., Shokouhian M., Ni S. (2001). Loading estimates of lead, copper, cadmium, and zinc in urban runoff from specific sources. Chemosphere.

[5] Stankovic S., Jovic M. (2011). Health risks of heavy metals in the mediterranean mussels as seafood. Environ. Chem. Lett.

[6] Pandard P., Vasseur P. (1992). Biocapteurs pour le contrôle de la toxicité des eaux: application des bioélectrodes algales. Rev. Sci. Eau/J. Water Sci.

[7] Giardi M.T., Koblízek M., Masojídek J. (2001). Photosystem II-based biosensors for the detection of pollutants. Biosens. Bioelectron.

[8] Naessens M., Leclerc J.-C., Tran-Minh C. (2000). Fiber optic biosensor using Chlorella vulgaris for determination of toxic compounds. Ecotoxicol. Environ. Safety.

[9] Nguyen-Ngoc H., Tran-Minh C. (2007). Fluorescent biosensor using whole cells in an inorganic translucent matrix. Anal. Chim. Acta.

[10] Védrine C., Leclerc J.-C., Durrieu C., Tran-Minh C. (2003). Optical whole-cell biosensor using Chlorella vulgaris designed for monitoring herbicides. Biosens. Bioelectron.

[11] Peña-Vázquez E., Maneiro E., Pérez-Conde C., Moreno-Bondi M.C., Costas E. (2009). Microalgae fiber optic biosensors for herbicide monitoring using sol-gel technology. Biosens. Bioelectron.

[12] Yang C.-F., Lee C.-M. (2008). Pentachlorophenol contaminated groundwater bioremediation using immobilized Sphingomonas cells inoculation in the bioreactor system. J. Hazard. Mater.

[13] Stormo K.E., Crawford R.L. (1992). Preparation of encapsulated microbial cells for environmental applications. Appl. Environ. Microbiol.

[14] Lestan D., Lamar R.T. (1996). Development of fungal inocula for bioaugmentation of contaminated soils. Appl. Environ. Microbiol.

[15] Bashan Y. (1986). Alginate beads as synthetic inoculant carriers for slow release of bacteria that affect plant growth. Appl. Environ. Microbiol.

[16] Arica M.Y., Kaçar Y., Genç O. (2001). Entrapment of white-rot fungus Trametes versicolor in Ca-alginate beads: preparation and biosorption kinetic analysis for cadmium removal from an aqueous solution. Bioresource. Technol.

[17] Nassif N., Bouvet O., Noelle Rager M., Roux C., Coradin T., Livage J. (2002). Living bacteria in silica gels. Nat. Mater.

[18] Amoura M., Nassif N., Roux C., Livage J., Coradin T. (2007). Sol–gel encapsulation of cells is not limited to silica: Long-term viability of bacteria in alumina matrices. Chem. Commun.

[19] Avnir D., Coradin T., Lev O., Livage J. (2006). Recent bio-applications of sol–gel materials. J. Mater. Chem.

[20] Livage J., Coradin T. (2006). Living cells in oxide glasses. Rev. Mineral. Geochem.

[21] Meunier C.F., Dandoy P., Su B.-L. (2010). Encapsulation of cells within silica matrixes: Towards a new advance in the conception of living hybrid materials. J. Colloid. Interface Sci.

[22] Nguyen-ngoc H., Tran-minh C. (2007). Sol-gel process for vegetal cell encapsulation. Mater. Sci. Eng.

[23] Blondeau M., Coradin T. (2012). Living materials from sol–gel chemistry: current challenges and perspectives. J. Mater. Chem.

[24] Brinker C.J., Scherer G.W. (1990). Sol-Gel Science: The Physics and Chemistry of Sol-Gel Processing.

[25] Léonard A., Dandoy P., Danloy E., Leroux G., Meunier C.F., Rooke J.C., Su B.-L. (2011). Whole-cell based hybrid materials for green energy production, environmental remediation and smart cell-therapy. Chem. Soc. Rev.

[26] Soltmann U., Böttcher H. (2008). Utilization of sol–gel ceramics for the immobilization of living microorganisms. J. Sol-Gel Sci. Technol.

[27] Darder M., Aranda P., Burgos-Asperilla L., Llobera A., Cadarso V.J., Fernandez-Sanchez C., Ruiz-Hitzky E. (2010). Algae-silica systems as functional hybrid materials. J. Mater. Chem.

[28] Perullini M., Jobbágy M., Soler-Illia G.J.A.A., Bilmes S.A. (2005). Cell Growth at Cavities Created Inside Silica Monoliths Synthesized by Sol-Gel. Chem. Mater.

[29] Boninsegna S., Dal Toso R., Dal Monte R., Carturan G. (2003). Alginate microspheres loaded with animal cells and coated by a siliceous layer. J. Sol-Gel Sci. Technol.

[30] Coradin T., Nassif N., Livage J. (2003). Silica-alginate composites for microencapsulation. Appl. Microbiol. Biotechnol.

[31] Perullini M., Rivero M.M., Jobbágy M., Mentaberry A., Bilmes S.A. (2007). Plant cell proliferation inside an inorganic host. J. Biotechnol.

[32] Sicard C., Perullini M., Spedalieri C., Coradin T., Brayner R., Livage J., Jobbagy M., Bilmes S.A. (2011). CeO_2_ Nanoparticles for the Protection of Photosynthetic Organisms Immobilized in Silica Gels. Chem. Mater.

[33] (1980). AFNOR, Détermination de l’inhibition de croissance de Scenedesmus subspicatus par une substance. Norme experimentale NT90-304.

[34] Gorman D.S., Levine R.P. (1965). Cytochrome f and plastocyanin: their sequence in the photosynthetic electron transport chain of Chlamydomonas reinhardi. Proc. Natl. Acad. Sci. USA.

[35] Nguyen-Ngoc H., Durrieu C., Tran-Minh C. (2009). Synchronous-scan fluorescence of algal cells for toxicity assessment of heavy metals and herbicides. Ecotoxicol. Environ. Saf.

[36] Perullini M., Jobbágy M., Bilmes S.A., Torriani I.L., Candal R. (2011). Effect of synthesis conditions on the microstructure of TEOS derived silica hydrogels synthesized by the alcohol-free sol–gel route. J. Sol-Gel Sci. Technol.

[37] Gasperi J., Garnaud S., Rocher V., Moilleron R. (2008). Priority pollutants in wastewater and combined sewer overflow. Sci. Total Envir.

[38] The European Parliament and the Council of the European Union. Directive 2008/105/EC of the European Parliament and the Council of 16 december 2008 on Environmental Quality Standards in the Field of Water Policy; 2008; pp. 84–97.

